# Incidence and Predictors of Surgical Site Infection in Patients Undergoing Coronary Artery Bypass Grafting at a Reference Hospital in Brazil: Influence of Sex, Nutritional Risk, and Body Mass Index

**DOI:** 10.21470/1678-9741-2025-0086

**Published:** 2026-02-18

**Authors:** Giovana Alves Carvalho, Julia Souza Siqueira de Andrade, Bruno Mahler Mioto, Luiz Aparecido Bortolotto

**Affiliations:** 1 Instituto do Coração (InCor), Faculdade de Medicina, Universidade de São Paulo (USP), São Paulo, São Paulo, Brazil

**Keywords:** Coronary Artery Bypass, Surgical Wound Infection, Postoperative Complications, Risk Factors.

## Abstract

**Introduction:**

Surgical site infection (SSI) following coronary artery bypass grafting (CABG) is a significant challenge that impacts quality of life and healthcare costs. Despite advances in surgical techniques and infection control measures, wound complications remain a major cause of morbidity and mortality. This study aimed to determine the incidence and factors associated with an increased risk of developing postoperative SSI in patients undergoing CABG.

**Methods:**

Retrospective cohort study with patients undergoing isolated CABG in a Brazilian hospital organization. Clinical data were collected through the hospital’s information system. Univariate and multivariate analyses were used to determine risk factors associated with SSI. The analyses were performed using Jamovi® software, with a significance set at P < 0.05.

**Results:**

A total of 412 patients were enrolled in the study, comprising 292 (70.8%) men, with a mean age of 62.7 ± 8.6 years. A total of 54 (13.1%) patients developed SSI. After multivariate regression analysis, the odds ratios (OR) (95% confidence interval [CI]) of independent predictors of SSI were female sex (OR: 2.067; 95% CI: 1.030 - 4.148), higher preoperative body mass index (OR: 1.113; 95% CI: 1.038 - 1.194), nutritional risk (Nutritional Risk Screening 2002 score ≥ 3) (OR: 2.468; 95% CI: 1.034 - 5.886), and hospitalization time (OR: 1.057; 95% CI: 1.031 - 1.082).

**Conclusion:**

There are patient-related factors that increase the likelihood of developing an SSI after CABG. These findings suggest that addressing modifiable perioperative SSI risk factors may be beneficial in reducing SSI rates and enhancing postoperative recovery.

## INTRODUCTION

**Table:** 

Abbreviations, Acronyms & Symbols	
BMI	= Body mass index
CABG	= Coronary artery bypass grafting
CAD	= Coronary artery disease
CI	= Confidence interval
CPB	= Cardiopulmonary bypass
ICU	= Intensive care unit
IQR	= Interquartile range
Md	= Median
NRS-2002	= Nutritional Risk Screening - 2002
OR	= Odds ratio
SSI	= Surgical site infection

Coronary artery disease (CAD), characterized by the buildup of atherosclerotic plaques within the arterial interior, remains a significant cause of morbidity and mortality both in Brazil and worldwide^[1]^. Treatment options for CAD vary depending on the severity of the condition and may include lifestyle changes, medication use, and more invasive procedures^[2]^. Conservative therapy focuses on symptom management, preventing disease progression, and minimizing the risk of adverse events, especially atherothrombotic events^[2,3]^. The clinical approach can be complemented by invasive therapy using percutaneous coronary intervention or coronary artery bypass grafting (CABG)^[2,3]^.

CABG is a crucial approach in the treatment of severe CAD, particularly beneficial in cases of greater anatomic complexity or when the patient has multiple comorbidities, and it is also the most frequently performed cardiac surgery^[4,5]^. However, even with advances in perioperative care, the complication rates associated with this procedure can reach up to 40%^[6]^. Among the most common complications, surgical site infections (SSI) frequently occur in individuals after cardiac surgery, with a reported incidence of 0.2% to 8.0%^[7]^, depending on factors such as the epidemiological profile of the population, presence of obesity, diabetes, chronic obstructive pulmonary disease, and prolonged duration of surgery^[5,7]^.

A post-procedure SSI can cause unnecessary physical and psychological pain, as well as prolonging hospital stays by up to 60%, generating a significant economic impact^[8]^. Additionally, indirect costs, such as the patient's temporary or permanent inability to perform their work duties, travel expenses, and home care costs, can be up to eight times higher than the direct costs associated with the infection^[9]^.

Finally, identifying risk factors for SSI is crucial for developing effective prevention, monitoring, and treatment strategies. Therefore, this study aims to analyze the incidence and determine the risk factors associated with SSI in patients undergoing CABG.

## METHODS

This is a retrospective, observational cohort study with a quantitative approach, based on the analysis of data extracted from a previously conducted clinical trial. The study was conducted at a single center, a reference institute for major surgery located in São Paulo, Brazil. The study was conducted in accordance with the Declaration of Helsinki, and the data collection occurred after approval by the Ethics Committee in Research, number 79980324.4.0000.0068, protocol number 7025215.

The study population consisted of patients who underwent CABG throughout 2023 and, in 2024, only those operated on between September and December, according to the original trial. Inclusion criteria encompassed patients aged 18 years or older who underwent an isolated elective surgical procedure within the specified period. Emergency surgeries and combined procedures were excluded.

The data were collected from the electronic medical records. The institution's Hospital Infection Control Center provided records of wound infections throughout the evaluation period. The data included sociodemographic variables (sex, age, and education level) and clinical variables (comorbidities, body mass index [BMI]), categorized according to the cutoff points established by the World Health Organization^[10]^ for adults and the Pan American Health Organization^[11]^ for patients over 60 years.

Additionally, preoperative nutritional risk was assessed using the Nutritional Risk Screening (NRS-2002)^[12]^, with patients scoring ≥ 3 considered at nutritional risk. Perioperative data were also recorded, including the type of graft, duration of surgery and cardiopulmonary bypass, length of stay in the intensive care unit (ICU), length of hospital stay, and incidence of SSI.

To analyze the data, quantitative variables were tested for normality and homogeneity using the Shapiro-Wilk test and Levene's test, respectively. Data with a normal distribution were presented as mean and standard deviation (±), while data with a non-normal distribution were presented as median and interquartile range. Categorical variables were expressed as absolute (n) and relative (%) frequencies. Student's *t*-test for independent samples or the Mann-Whitney U test were used to compare quantitative variables, depending on data normality. Associations between categorical variables were analyzed using Pearson's chi-square test, Pearson's chi-square test with Yates' correction, or Fisher's exact test.

To assess factors associated with SSI development, a univariate analysis was conducted using logistic regression. Next, variables with a *P*-value < 0.20 were included in a multivariate regression model, with their estimators analyzed using the odds ratio (OR) as the measure of association, in which the assumptions of multicollinearity and homoscedasticity were respected, with analysis of the variance inflation factors close to the value 1 and tolerance between the independent variables > 0.8. Factors with high association or correlation were excluded from the final multivariate model.

A 95% confidence interval (CI) was used, with a *P*-value < 0.05 considered statistically significant. Effect size was evaluated using Cohen's d, Cramer's V, and biserial correlation of ranks. Data analysis was performed using Jamovi 2.2.5®.

## RESULTS

A total of 412 patients were included in this study. Of these, 54 developed an SSI (13.1%). Table 1 presents the sociodemographic and clinical characteristics of patients in each group.

**Table 1:** Sociodemographic and clinical characteristics of patients undergoing coronary artery bypass grafting, stratified by incidence of surgical site infections (SSI), in São Paulo, Brazil, 2023-2024.

Clinical and sociodemographic data	SSI		Total	Statistical test
	No	Yes	(N = 412)	
	(N = 358)	(N = 54)		
Male, n (%)	265/358 (74.0%)	27/54 (50.0%)	292/412 (70.8%)	χ2(1) = 13.1, *P* < 0.01^1^
Age, mean (±)	62.4 (8.6)	64.9 (7.9)	62.7 (8.6)	t(411) = 2.00, *P* = 0.04^4^
Age group, n (%)				
< 60 years	120/358 (33.5%)	11/54 (20.3%)	131/412 (31.7%)	χ2(1) = 3.74, *P* = 0.05^1^
≥ 60 years	238/358 (66.4%)	43/54 (79.6%)	281/412 (68.2%)	
Education level, n (%)				
Unknown	19/358 (5.3%)	4/54 (7.0%)	23/412 (5.5%)	χ2(2) = 4.42, *P* = 0.49^2^
None	6/358 (1.6%)	2/54 (3.7%)	8/412 (1.9%)	
Elementary	194/358 (54.1%)	33/54 (61.1%)	227/412 (55.0%)	
High school	94/358 (26.2%)	8/54 (14.8%)	102/412 (24.7%)	
College degree	45/358 (12.5%)	7/54 (12.9%)	52/412 (12.6%)	
Comorbidities, n (%)				
Diabetes mellitus	174/358 (48.6%)	35/54 (64.8%)	209/412 (50.7%)	χ2(1) = 4.93, *P* = 0.02^1^
Hypertension	306/358 (85.4%)	48/54 (88.8%)	354/412 (85.9%)	χ2(1) = 0.45, *P* = 0.50^1^
Renal impairment	24/358 (6.7%)	7/54 (12.9%)	31/412 (7.50%)	χ2(1) = 2.64, *P* = 0.10^1^
BMI, Md (IQR)	27.4 (24.7-30.0)	29.9 (25.0-34.0)	27.5 (24.7-30.4)	U = 7411, *P* < 0.01^3^
Stratified BMI, n (%)				
Underweight	40/358 (11.1%)	5/54 (9.2%)	45/412 (10.9%)	
Normal weight	164/358 (45.8%)	16/54 (29.6%)	180/412 (43.6%)	χ2(1) = 11.6, *P* < 0.011
Overweight	67/358 (18.1%)	8/54 (14.8%)	75/412 (18.2%)	
Obese	87/358 (24.3%)	25/54 (46.2%)	112/412 (27.1%)	
NRS 2002, n (%)				
Nutritionally at-risk	33/358 (9.2%)	13/54 (24.0%)	46/412 (11.1%)	χ2(1) = 10.4, P < 0.01^1^
Grafting, n (%)				
Left internal thoracic artery	354/358 (98.8%)	53/54 (98.1%)	407/412 (98.7%)	χ2(1) = 0.21, *P* = 0.64^1^
Right internal thoracic artery	45/358 (12.5%)	6/54 (11.1%)	51/412 (12.3%)	χ2(1) = 0.09 *P* = 0.76^1^
Radial artery	154/358 (43.0%)	15/54 (27.7%)	169/412 (41.0%)	χ2(1) = 4.50, *P* = 0.03^1^
Saphenous vein	299/358 (83.5%)	43/54 (79.6%)	342/412 (83.0%)	χ2(1) = 0.50, *P* = 0.47^1^
Surgery time (hours), Md (IQR)	4.8 (4.2 - 5.6)	5.2 (4.7 - 5.6)	5.0 (4.2 - 5.6)	U = 7722, *P* = 0.01^3^
CPB time (minutes), Md (IQR)	95.0 (74.0 - 110.0)	96 (85.0 - 109.0)	95.0 (75.0 - 110.0)	U = 7288, *P* = 0.44^3^
ICU length of stay (days), Md (IQR)	2.8 (2.0 - 3.8)	3.9 (2.2 - 7.8)	2.8 (2.0 - 4.0)	U = 7004, *P*< 0.01^3^
Hospitalization time (days), Md (IQR)	8.2 (7.1 - 11.1)	15.0 (9.2 - 29.9)	8.9 (7.2 - 12.2)	U = 4581, *P*< 0.001^3^

1 Pearson’s chi-square test,

2 Pearson’s chi-square test with Yates’ correction,

3 Mann-Whitney U test,

4 Student’s *t*-test

BMI=body mass index; CPB=cardiopulmonary bypass; ICU=intensive care unit; IQR=interquartile range; Md=median; NRS=Nutritional Risk Screening

Most study participants were male (n = 292; 70.8%) with a mean age of 62.7 ± 8.6 years, showing a significant difference between groups (*P* = 0.04). There was a predominance of diabetic patients (n = 35; 64.8%; *P* = 0.02) and those with renal impairment (n = 7; 12.9%; *P* < 0.01) in the SSI group. The BMI was also significantly higher among patients with SSI (*P* < 0.01). The NRS-2002 indicated that among the patients, there were greater numbers of participants at nutritional risk in the group with SSI (*P* < 0.01). Among the grafts used, only the use of the radial artery differed significantly between the groups (*P* = 0.03). Surgery time (*P* = 0.01), ICU length of stay (*P* < 0.01), mean postoperative hospital stay (*P* < 0.001), and total length of hospital stay (*P* < 0.001) were all longer in the SSI group.

The risk factors associated with SSI following CABG surgery for all patients using univariate regressions are presented in Table 2. Those who developed an SSI were more likely to be female patients (OR: 2.438; 95% CI: 1.358 - 4.375), patients aged ≥60 years (OR: 2.266; 95% CI: 1.102 - 4.658), diabetics (OR: 1.937; 95% CI: 1.068 - 3.515), in renal impairment (OR: 2.413; 95% CI: 1.024 - 5.671), to have a high BMI (OR: 1.092; 95% CI: 1.027 - 1.162), at nutritional risk (OR: 3.103; 95% CI: 1.512 - 6.372), with high surgery time (OR: 1.416; 95% CI: 1.082 - 1.855), and longer ICU length of stay (OR: 1.045; 95% CI: 1.014 - 1.078) and hospitalization time (OR: 1.061; 95% CI: 1.039 - 1.084). One factor associated with a decreased risk of developing SSI was patients who received radial graft (OR: 0.459; 95% CI: 0.241 - 0.874).

**Table 2:** Univariate analysis showing the association between risk factors and odds of a surgical site infection in patients undergoing coronary artery bypass grafting, in São Paulo, Brazil, 2023-2024.

Variables	*P*-value	Odds ratio	95% CI	
			Lower	Upper
Sex				
Female - male	0.003	2.438	1.358	4.375
Age group				
< 60 years - ≥ 60 years	0.026	2.266	1.102	4.658
Education level				
None - unknown	0.522	1.900	0.267	13.523
Elementary - unknown	0.721	0.812	0.260	2.539
High school - unknown	0.171	0.404	0.110	1.480
College degree - unknown	0.658	0.739	0.193	2.823
Diabetes mellitus				
Yes - no	0.031	1.937	1.068	3.515
Hypertension				
Yes - no	0.494	1.368	0.557	3.360
Renal impairment				
Yes - no	0.044	2.413	1.024	5.671
Preoperative BMI (kg/m^2^)	0.005	1.092	1.027	1.162
Stratified BMI				
Normal weight - underweight	0.670	0.792	0.272	2.313
Overweight - underweight	0.302	0.542	0.169	1.736
Obese - underweight	0.368	1.620	0.567	4.632
NRS 2002				
At risk - not at risk	0.002	3.103	1.512	6.372
Left internal thoracic artery				
Yes - no	0.653	0.602	0.066	5.492
Right internal thoracic artery				
Yes - no	0.751	0.864	0.350	2.134
Radial artery				
Yes - no	0.018	0.459	0.241	0.874
Saphenous vein				
Yes - no	0.490	1.288	0.628	2.642
Surgery time (hours)	0.011	1.416	1.082	1.855
CPB time (minutes)	0.983	1.000	0.989	1.012
ICU length of stay (days)	0.004	1.045	1.014	1.078
Hospitalization time(days)	< 0.001	1.061	1.039	1.084

BMI=body mass index; CI=confidence interval; CPB=cardiopulmonary bypass; ICU=intensive care unit; NRS=Nutritional Risk Screening

Using the variables that were found to have a significant association in Table 2, a multivariable logistic regression model was developed to determine risk factors associated with development of SSI (Table 3). Being a woman doubled the risk of infection (OR: 2.067; 95% CI: 1.030 - 4.148). Each one-unit increase in BMI was associated with an 11% higher SSI risk (OR: 1.113; 95% CI: 1.038 - 1.194). Nutritional risk (OR: 2.468; 95% CI: 1.034 - 5.886) and longer hospitalization time (OR: 1.057; 95% CI: 1.031 - 1.082) were also significant factors for increased SSI risk.

**Table 3:** Multivariate regression model showing the association between risk factors and odds of a surgical site infection in patients undergoing coronary artery bypass grafting, in São Paulo, Brazil, 2023-2024.

Variables	*P*-value	Odds ratio	95% CI	
			Lower	Upper
Sex				
Female - Male	0.041	2.067	1.030	4.148
Age group				
< 60 years - ≥ 60 years	0.350	0.665	0.282	1.564
Education level				
None - unknown	0.363	0.214	0.007	5.908
Elementary - unknown	0.348	0.549	0.157	1.917
High school - unknown	0.112	0.308	0.072	1.313
College degree - unknown	0.761	0.794	0.179	3.513
Diabetes mellitus				
Yes - no	0.172	1.613	0.811	3.206
Renal impairment				
Yes - no	0.596	0.718	0.211	2.441
Preoperative BMI (kg/m^2^)	0.003	1.113	1.038	1.194
NRS 2002				
At risk - not at risk	0.042	2.468	1.034	5.886
Radial artery				
Yes - no	0.267	0.660	0.316	1.375
Surgery time (hours)	0.459	1.126	0.822	1.107
Hospitalization time(days)	<0.001	1.057	1.031	1.082

BMI=body mass index; CI=confidence interval; NRS=Nutritional Risk Screening

## DISCUSSION

In this retrospective cohort study rates of and risk factors for SSI among patients undergoing CABG were identified. Results showed that the incidence rate of SSI after CABG surgeries in the evaluated center is 13.1%. Nationally, the current study showed that the infection rate is less than the reported rates from Rio Grande do Sul (19.1%)^[13]^ and Minas Gerais (19.0%)^[8]^. At the international level, most of the multicenter studies have reported incidence rates much lower than the rate reported in the current study. For example, the overall incidence rate of SSI after CABG surgeries in Sweden is 1.3%^[14]^ and in Italy, it is 2.4%^[15]^. Likewise, the incidence rate in studies from the United States of America is 3.7%^[16]^.

Results demonstrated that the odds of an SSI were significantly higher in females even after adjusting for risk factors using multivariate regression. Sex inequalities in SSIs have been firmly established, and our figures align with the literature^[17,18]^. A risk prediction model from the United Kingdom highlights female sex as one of six independent factors associated with SSI after cardiac surgery^[9]^. This disparity may partially be attributed to the fact that females undergoing CABG are often older and have more comorbidities than males, as coronary disease is recognized later in women, leading to delays in diagnosis and treatment^[19]^. Also, hormonal factors and the proximity of breast tissue create inferolateral tension on the incision, increasing the likelihood of wound-healing disturbances in women and potentially providing a medium for microbial growth^[20]^.

In this study, BMI category was not significantly associated with SSI in the univariable analysis. However, in multivariable regression modeling, a trend of increasing risk of SSI was observed for each additional unit increase in BMI. One of the reasons for the higher risk of infections after surgery in overweight patients is the hypoperfusion of adipose tissue, which can lead to delayed wound healing and the formation of dead space, increasing the likelihood of local tissue necrosis and, consequently, the entry and proliferation of microorganisms^[21]^. Overweight patients are also more prone to comorbidities, especially diabetes, hypertension, and metabolic syndrome, which can indirectly favor the onset or worsening of SSI^[21]^.

In this survey, 11.1% of patients had nutritional risk before surgery according to the NRS 2002 criteria, and preoperative nutritional risk was strongly correlated with increased rates of SSI. This tool is based on five variables: weight loss, BMI, amount of food intake in the preceding week, patients’ age, and the severity of the underlying disease^[12]^. It is recognized as a more reliable preoperative nutritional screening score compared to other tools^[22]^. Similar to the present findings, other studies have also confirmed that nutritional risk is a significant reason for infection. Zhang et al.^[22]^ found that the NRS 2002 could predict SSI after abdominal surgery. Shang et al. assessed the impact of nutritional risk using the same instrument in patients undergoing total joint arthroplasty, and their results were associated with increased SSI-related readmission^[23]^. Therefore, it is important to assess the nutritional risk using preoperative NRS 2002 score for prediction of SSI.

A prolonged length of stay was also significantly associated with the presence of SSI. While an increased length of stay may result from the need for SSI treatment, extended hospital stays due to the management of other in-hospital complications could also elevate the risk of developing SSI, as the hospital environment may contribute to the microbiological contamination of surgical wounds^[24]^. As a result of this complication and the increased length of stay in hospital, hospitals can be hit with increased costs for the management and a shortage of available bed space for the management of new patients^[25]^.

### Limitations

This study has several limitations. First, the observational and retrospective design has its own shortcomings, such as follow-up bias. Second, the data came from a single health center, and the heterogeneity of clinical practice between hospitals may limit the relevance of the findings. Third, some possible risk factors for the development of SSI were excluded from the evaluation due to the inaccessibility of the information in the electronic medical records.

## CONCLUSION

In conclusion, this study identified the incidence and risk factors for SSI in patients undergoing CABG. Patient-related factors associated with SSI were identified: infection occurred more frequently in patients who were female, had a higher BMI, were at nutritional risk, and had a longer hospital stay. SSIs are complex events, and the identification of modifiable risk factors requires a multidisciplinary effort to decrease rates and improve postoperative outcomes.

## Data Availability

The authors declare that the data will be available upon request to the authors.

## Artificial Intelligence Usage

The authors declare that no artificial intelligence tool was used in the preparation of this article.
